# Ocular Surface Squamous Neoplasia: A 12-Month Prospective Evaluation of Incidence in Waikato, New Zealand

**DOI:** 10.3390/vision6030050

**Published:** 2022-08-12

**Authors:** Ruhella R Hossain, Jee Ah Oh, Cameron McLintock, Chris Murphy, James McKelvie

**Affiliations:** 1 Department of Ophthalmology, Faculty of Medical and Health Sciences, University of Auckland, Auckland 1010, New Zealand; 2 Department of Ophthalmology, Hawkes Bay District Health Board, Hastings 4120, New Zealand; 3 Department of Ophthalmology, Princess Alexandra Hospital, Brisbane, QLD 4102, Australia; 4 Department of Ophthalmology, Waikato District Health Board, Hamilton 3204, New Zealand

**Keywords:** ocular surface squamous neoplasia, OSSN, epidemiology

## Abstract

Ocular surface squamous neoplasia (OSSN) has a high incidence in the southern hemisphere. This prospective study evaluated the incidence of OSSN in the Waikato region of New Zealand. All patients presenting with pterygium or conjunctival lesions in the Waikato region in 2020 were included. All surgeons in the region were asked to send all conjunctival and corneal specimens excised for histopathologic examination. The primary outcome measure was the incidence of OSSN. Eighty-eight percent of all excised specimens were sent for histopathologic examination. Of the 185 excised lesions sent for histopathological assessment, 18 (10%) were reported as OSSN. Patients were on average 69.4 years of age (standard deviation, SD = 6.9), predominantly male (78%), and of New Zealand-European ethnicity (89%). The OSSN annual incidence was 3.67/100,000/year. Histology grades included conjunctival intraepithelial neoplasia (CIN)-I (25%), CIN-II (25%), CIN-III (12.5%), carcinoma in situ (25%), and invasive squamous cell carcinoma (SCC) (12.5%). One patient with invasive SCC required exenteration. This study identified a high incidence rate of OSSN and is the first prospective study to analyze OSSN epidemiology in New Zealand.

## 1. Introduction

Ocular surface squamous neoplasia (OSSN) refers to a spectrum of abnormal growth of atypical squamous epithelial cells of the conjunctival, limbal, and corneal epithelium [1,2,3,4,5,6,7]. Histopathology classification of lesions include conjunctival intraepithelial neoplasia (CIN) grades I–III, carcinoma in situ (CIS), or invasive squamous cell carcinoma (SCC) depending on the extent of penetration [3,4]. Mild dysplasia, or CIN grade I, involves the lower third of the epithelial thickness; moderate dysplasia, or CIN grade II, involves the lower two-thirds; severe dysplasia, or CIN grade III, involves more than two-thirds thickness; and CIS involves the entire epithelium. Invasive SCC occurs when the dysplastic epithelial cells penetrate the corneal basement membrane and spread into the conjunctival stroma.

Incidence rates of OSSN vary globally depending on geography and risk factors [3,4,8,9,10]. Higher incidence rates of OSSN exist in the southern hemisphere compared to the northern hemisphere [11]. The reported rate for OSSN incidence varies from 0.053/100,000/year in the United Kingdom to 2.8/100,000/year in Brisbane, Australia [8,10]. Risk factors for OSSN include older age, ultraviolet light exposure, fair skin pigmentation, and immunocompromised status [4,11,12,13]. Similar risk factors are reported in the United Kingdom [10], the United States of America [14], and Canada [9].

The Waikato region in New Zealand has one of the highest reported rates of OSSN [15]. A recently published retrospective study of OSSN patients in the Waikato region reported an incidence of 2.13/100,000/year with a peak annual OSSN incidence of 3.81/100,000/year in 2019 [15]. The aim of this prospective study was to validate the retrospective study and evaluate the regional incidence of OSSN in 2020.

## 2. Materials and Methods

This prospective study adhered to the tenets of the Declaration of Helsinki and New Zealand Ethical Guidelines for Observational Studies [16]. Ethical approval was obtained from the Auckland District Health Board Research Review Committee (AH24384).

All public and private sector surgeons performing ophthalmic surgery in the Waikato region were asked to send all excised conjunctival, corneal, and scleral cysts, masses or lesions, including suspected OSSN specimens in 2020 for histological analysis. Operating theatre records were reviewed from all public and private hospitals in the region that complete ophthalmic surgery. Patients who underwent removal, incision, or excision biopsy of any conjunctival lesion, including clinically suspected pterygia, and had histological grading of the specimen in 2020 were included. Each histology report was reviewed for OSSN grade and presence at surgical margins.

The primary outcome measure was the number of patients with histologically confirmed OSSN that occurred for the first time in that patient. The secondary outcome measure was patient demographic variables for those with primary OSSN, including age at the time of surgery, sex, and self-reported ethnicity.

All data analyses were completed in R Version 3.5.2 (R Foundation for Statistical Computing, Vienna, Austria). The OSSN regional incidence rates were defined as the annual number of new cases per 100,000 population per year using population estimates from the New Zealand 2018 National Census [17].

## 3. Results

In 2020, there were 210 patients who had a surgical excision of conjunctival, corneal, or scleral lesions in the Waikato. These 210 patients were mostly male (n = 113, 54%) with an average age of 58.0 years (SD 16.2). Patient ethnicities included New Zealand-European (n = 74, 35%), Māori (n = 13, 6%), Asian (n = 9, 4%), Pasifika (n = 6, 3%), and Other/Not stated (n = 108, 51%).

Preoperative diagnoses listed for all 210 lesions included suspected pterygia (n = 174, 82.9%), suspected OSSN including SCC (n = 17, 8.1%), pinguecula (n = 11, 5.2%), and cyst/naevus/other (n = 8, 3.8%). Preoperative data was not available on the extent of corneal or scleral involvement. No patient received preoperative chemoreduction. Adjunctive cryotherapy was used for five (29.4%) of the 17 preoperatively suspected OSSN cases.

Eye laterality was similar for all excised lesions (left eye n = 106, 50.5%; right eye n = 103, 49.0%; unknown n = 1, 0.5%). Information on excised tissue location was available for 76 lesions (36%) and included nasal only n = 65 (85.5%), nasal and temporal n = 6 (7.9%), temporal only n = 4 (5.3%), and inferotemporal only n = 1 (1.3%).

In total, 185 (88%) samples were sent for histopathological analysis during the study period. The remaining 25 samples either were not sent for histopathological analysis or the results could not be located. These 25 samples were diagnosed as clinically suspected pterygia.

Postoperative diagnoses for all 185 histopathology reports included OSSN (n = 18, 10%), pterygium (n = 135, 73%), and other (n = 32, 17%). Incomplete marginal excision was reported in two samples. The preoperative diagnoses of all 18 histologically confirmed OSSN cases included suspected OSSN (n = 13, 72%), pterygia (n = 4, 22%), and pinguecula (n = 1, 6%).

Two patients diagnosed with histologically confirmed OSSN had a repeat biopsy performed two months after their initial surgery, leaving 16 patients with newly diagnosed OSSN. These 16 patients were mostly male (n = 12, 75%) and had an average age of 69.4 years (SD 6.9). Ethnicities included New Zealand-European (n = 14, 88%), Māori (n = 1, 6%), and Other/Not stated (n = 1, 6%).

In 2020, the Waikato region population included 435,690 people [17]. The annual incidence of OSSN was 3.67/100,000/year in 2020. Histology grades of each identified OSSN specimen and average age of patients are shown in Table 1.

All 16 patients with histologically confirmed OSSN postoperatively received either one cycle of 0.02% mitomycin-C (MMC) four times a day for two weeks or three cycles of 0.04% MMC four times a day with one week on and one off, according to surgeon preference. One of the two patients with invasive SCC developed severe pain secondary to local spread involving the lacrimal gland. This patient underwent exenteration for pain management and to prevent further spread. None of the other patients developed recurrence or treatment-related complications. All cases of OSSN will remain under follow-up for a minimum of three years to monitor for recurrence.

## 4. Discussion

This is the first prospective study to assess the incidence of histologically diagnosed OSSN in New Zealand. All ophthalmic surgeons in the Waikato region participated in the study. There were 185 excised lesions sent for histopathological assessment with 10% diagnosed as OSSN. The patients were mostly Caucasian (88%) or male (75%).

Clinical diagnosis of OSSN lesions without histology can be challenging. In this study, four (23.5%) of the 17 preoperatively suspected OSSN lesions were histologically diagnosed as pterygia (n = 2, 11.8%), lymphoma (n = 1, 5.9%), or chronic inflammation (n = 1, 5.9%). Conversely, 13 (72.2%) of the 18 histologically confirmed OSSN lesions were preoperatively suspected as OSSN. The remaining five histologically confirmed OSSN lesions were preoperatively suspected pterygia (n = 4, 22.2%) and pinguecula (n = 1, 5.6%). Diagnostic adjuncts can be useful, including vital dye staining, cytology, in vivo confocal microscopy, and anterior segment-optical coherence tomography (AS-OCT) [18,19,20,21,22,23,24,25,26,27]. However, each modality comes with its own limitations, and caution must be used in interpreting these tests. For example, vital dye stains are not specific for OSSN, and benign lesions can stain positive in some cases [28,29]. Aspiration or impression cytology have shown good histological correlation but require an adequate sample and pose a risk of false negative results if used for diagnostic purposes [23,28,29,30,31,32]. Case reports highlight the utility of in vivo confocal microscopy and AS-OCT but discuss the difficulty in reliably distinguishing OSSN and benign lesions [22,25,33,34,35]. While these alternative modalities are valuable tools for suspected cases and monitoring, incisional or excisional biopsy remains the gold standard for diagnosis. Chronic and cumulative exposure to ultraviolet radiation is a risk factor for OSSN, a condition where lesions have a predilection for sun-exposed locations on the eye [12]. Cumulative and chronic ultraviolet radiation is associated with DNA damage and reactivation of human papillomaviruses (HPV), both known risk factors for OSSN [11,12,36]. In the current study, information on the location of the excised lesion was available for 36% of all 210 excised lesions. Of these, all were located within regions susceptible to sun exposure.

This one-year prospective study of histologically analyzed specimens reports one of the highest global incidence rates of OSSN (3.67/100,000/year). A recently published 10-year retrospective study of patients in the Waikato region reported an estimated 2.13/100,000/year with a peak annual OSSN incidence of 3.81/100,000/year in 2019 [15]. However, in that study, only 33% of the excised lesions in the Waikato region had available histopathology reports. It was not clear if selection bias had skewed the study results in this retrospective study, and further validation was required. The current prospective study was performed in the same locations with histopathology reports available for 88% of all excised lesions. The high rate of OSSN identified in this 2020 prospective study confirms the previously reported rates. Clinicians should maintain a high degree of suspicion for OSSN, especially in patients with multiple risk factors.

All OSSN patients identified in this prospective study received surgical excisional biopsy of the lesion followed by postoperative adjuvant chemotherapeutic MMC at either 0.04% or 0.02%, according to surgeon preference. No patient received preoperative chemotherapeutic agents. Topical therapy can be used in isolation without surgical excision; however, in the current study, surgeon preference was excision biopsy with histology to confirm the diagnosis. There is a mixed consensus in the literature on whether or not to institute preoperative therapy for suspected OSSN lesions [37,38]. Standard-of-care surveys administered in the United States in 2003 and 2012 reported that 51% of respondents always performed biopsies before initiating therapy for suspected OSSN lesions [37,38]. However, a prospective multicenter study in Kenya found that OSSN and benign conjunctival lesions could not reliably be distinguished clinically, suggesting caution in instituting preoperative therapy [35].

Topical therapy for OSSN includes MMC, interferon-ɑ2b (IFN), or 5-fluorouracil (5FU) [27,39,40,41,42]. Topical therapy will treat the entire ocular surface and is generally tolerated well. Side effects include punctal stenosis, limbal stem cell deficiency, conjunctivitis, lid toxicity, epithelial microcysts, and corneal erosion or ulceration [27,40]. These side effects are rare and can be minimized or avoided with the use of punctal plugs and nonpreserved lubricants during the treatment phase [27]. Regular review is required with topical therapy to monitor for adverse reactions and adherence during the treatment period.

In this prospective study, two patients required a repeat excision biopsy two months after their initial biopsy. In these cases, incomplete marginal excisions occurred. OSSN predominantly recurs within the first two years with increased risk from incomplete margins [7,43]. Decreased recurrence rates are reported with a “no-touch” surgical excision technique with wide margins and cryotherapy or postoperative adjuvant therapy [7,43,44]. The results of this study highlight the need for routine histology for any excised conjunctival lesion. Ongoing monitoring of biopsy-proven cases of OSSN is advisable to detect early recurrences. All histological grades of OSSN were identified in the current study cohort and, apart from one patient, all patients responded well to MMC treatment with a low rate of recurrence. One patient with invasive SCC developed severe ocular pain and a widespread ocular surface tumor with local spread to the lacrimal gland.

Limitations of this prospective non-interventional study include lack of information on immunocompromised status and data on preoperative clinical symptoms. However, qualifying criteria for a surgical excision or biopsy of an ocular lesion at the Waikato public-funded hospital included clinical suspicion of OSSN, lesion encroaching on the visual axis or significant induced corneal astigmatism, or significant clinical symptoms including pain, irritation, or chronic redness.

This is the first prospective study analyzing the epidemiology of OSSN in New Zealand’s Waikato region. OSSN typically responds well to treatment, and the mortality rate is very low when the disease is identified early. Delays in diagnosis or treatment can lead to preventable loss of vision and the need for more aggressive treatment including exenteration. Routine histological analysis of all conjunctival specimens is important to accurately diagnose, treat, and monitor OSSN.

## Figures and Tables

**Table 1:** Histology grades and age of patient at time of OSSN diagnosis. CIN = conjunctival intraepithelial neoplasia. CIS = carcinoma in situ. SCC = squamous cell carcinoma.

Histology Grade	Number (%)	Average Age (SD)
CIN grade I	4 (25)	67 (11.0)
CIN grade II	4 (25)	72.5 (6.9)
CIN grade III	2 (12.5)	72.5 (4.9)
CIS	4 (25)	69.75 (2.1)
Invasive SCC	2 (12.5)	62.5 (4.9)
TOTAL	16 (100)	69.4 (6.9)

## Data Availability

Data is contained within the article.
